# Predictors of activities of daily living in heathy older adults: Who benefits most from online cognitive training?

**DOI:** 10.1002/brb3.2388

**Published:** 2021-10-17

**Authors:** Mandy Roheger, Elke Kalbe, Anne Corbett, Helen Brooker, Clive Ballard

**Affiliations:** ^1^ Department of Medical Psychology Neuropsychology and Gender Studies & Center for Neuropsychological Diagnostics and Intervention (CeNDI) Faculty of Medicine and University Hospital Cologne Cologne Germany; ^2^ Department of Neurology University Medicine Greifswald Greifswald Germany; ^3^ Institute of Health Research University of Exeter Medical School University of Exeter Exeter UK

**Keywords:** healthy older adults, prediction, reasoning cognitive training

## Abstract

**Objectives:**

To investigate the course of activities of daily living (IADL) functioning and possible predictors of performance changes in healthy older adults conducting either a General Cognitive Training (GCT) or a Reasoning Cognitive Training (ReaCT) or no training (control group, CG) over a period of 6 weeks, 3 months, and 6 months.

**Setting and participants:**

An online, home‐based GCT and ReaCT including *n* = 2913 healthy participants (GCT: *n* = 1096; ReaCT: *n* = 1022; CG: *n* = 794) aged 60 years and older.

**Methods:**

Multilevel analysis were calculated to explore the nature of our outcome variables of IADL part A (independence) and part B (difficulty of tasks), and to detect possible predictors for participants’ performance on IADL after CT.

**Results:**

The random slopes models fitted better for the outcomes IADL Part B in the GCT group (χ^2^(2) =  18.78, *p* < .01), and both IADL Part A and Part B in the ReaCT group (χ^2^(2) =   28.57, *p* < .01; χ^2^(2) =   63.38, *p* < .01, respectively), indicating different changes over time for individual participants. Female sex was a significant predictor of IADL change in the ReaCT group, showing that females benefited most in both IADL scores (IADL A: 0.01, *p* < .01; IADL B: 0.004, *p* < .01). No other significant predictors for IADL changes were identified.

**Conclusion and implication:**

The particular effectiveness in women is of clinical relevance, as IADL is typically more impaired in women than in men in advanced age. Following a personalized medicine approach, identifying predictors of non‐pharmacological intervention success is of utmost importance.

## Graphical abstract

This is the first study investigating possible predictors for change of instrumental activities of daily living (IADL) performance during two different online cognitive trainings (CT, General CT and Reasoning CT). While previous analysis had shown that CT generally may enhance IADL, our results show that being female predicts improvements in IADL independence and difficulty, but only when conducting Reasoning CT. Following a personalized medicine approach, this result is of high importance in designing further CTs and fitting specific CTs to participant s' needs.

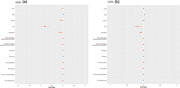

## 1 INTRODUCTION

As we age, our cognitive abilities decline, leading amongst others to increased difficulty in performing instrumental activities of daily living (IADL) (Kelly‐Hayes et al., 1992; Stuck et al., 1999; Wolinsky et al., 1993). IADL includes activities such as using public transportation, managing finances, or shopping (Lawton & Brody, 1969) that require complex neuropsychological processing capacity and are prone to deterioration by a cognitive decline (Agüero‐Torres et al., 2002). Notably, dysfunction in IADL is also a hallmark of dementia and a strong predictor of progression to dementia in patients with mild cognitive impairment (MCI) (Di Carlo et al., 2016).

Systematic reviews and meta‐analysis show that one possible way to maintain or improve cognitive abilities is cognitive training (CT) (Chiu et al., 2017; Joubert & Chainay, 2018). CT can also help to improve IADL abilities in healthy older participants (Fan & Wong, 2019; Rebok et al., 2014) and patients with MCI (Liao et al., 2020). As maintaining functional independence is of great importance for older adults (Feger et al., 2020) and is associated with increased quality of life (Andersen et al., 2004) and lower health care expenditures (Liu et al., 1997), it is not only important to know which CT is (on a group level) the most effective in increasing IADL, but also which factors (e.g., sociodemographic variables, (neuro‐)psychological variables, genetic and brain imaging parameters) determine responsiveness to CT. Regarding prediction of IADL, walking speed, memory, and processing speed have been shown to independently predict IADL limitation in older adults without cognitive impairment (Burton et al., 2018; Makizako et al., 2015), and better performance in attention/processing speed and executive functioning predicted IADL in patients with MCI (Putcha & Tremont, 2016). Regarding prediction of CT responsiveness, sociodemographic variables (e.g., age, sex, education), neuropsychological baseline scores at test entry, brain imaging parameter, personality traits, mood, and genetic variables have been factors identified to predict cognitive change (Roheger et al., 2020). However, to the authors’ best knowledge, no studies exist investigating predictors of IADL responsiveness to CT in healthy adults—even though this knowledge could help, for example, in the process of decision‐making to a specific CT. In a previous analysis of the present data set, we re‐analyzed data of a large RCT in which effects of two digital CTs (general cognitive training [GCT] or reasoning cognitive training [ReaCT]) were compared to those of a passive Control Group [CG] (Corbett et al.,^2015^ ). In the first step, we investigated predictors of cognitive outcomes showing that being female was predictive for improvement in grammatical reasoning at 6 weeks, and lower cognitive baseline scores were predictive for improvement in spatial working memory and verbal learning at 6 months (Roheger et al., 2020a) In the ReaCT group, being female and having lower education predicted improvements in grammatical reasoning scores at 6 weeks and 3 months of training (Roheger et al., 2020b). IADL data was not included in the previous prediction analysis for several reasons. First, difficulties in IADL typically become apparent in an early clinical phase of dementia, so that the outcome—as a clinical sign—fundamentally differs from cognitive measures in healthy aging. Second, and related to this, IADL in the study we refer to was only assessed for participants older than 60 years, whereas all other outcomes were assessed for participants older than 50 years, also resulting in different sample sizes for these different outcomes. Finally, it should be noted that to the authors’ best knowledge, no studies investigate predictors of IADL performance while conducting a CT exist.

Therefore, we aim to investigate what variables of our previous study (namely age, sex, education, level of depressive symptoms, time, and number of intervention sessions) predict individual IADL performance. For this purpose, we re‐analyzed data of a large RCT in which effects of two digital CTs (general cognitive training [GCT] or reasoning cognitive training [ReaCT]) were compared to those of a passive Control Group [CG] (Corbett et al., 2015). The analysis was conducted in two steps: first, the nature of performance of our outcome variables was investigated (meaning: which mathematical model fits best to explain the course of our data) to determine a model for our prediction analysis, which was conducted in a second step. As this is an exploratory post hoc data analysis and no data on predictors of IADL responsiveness to CT in healthy adults exist, we did not state any specific a‐priori hypothesis. However, we believe that the integrated predictor's age, sex, education, level of depressive symptoms, time, and number of intervention sessions may have a substantial influence on IADL training performance.

## 2 METHODS

### 2.1 Study design

This is a post hoc data analysis of an already published study, that is, a double‐blind 6‐month online randomized three‐arm controlled trial (GCT vs. ReaCT vs. active Control Group [CG]) with healthy adults older than 60 years conducted in the United Kingdom (GCT: *n* = 1096; ReaCT: *n* = 1022; CG: *n* = 794). The study comprised four measurement times: Baseline, 6 weeks, 3 months, and 6 months. IADL was measured using the Minimum Data Set‐Home Care IADL scale, which is based on four levels of self‐performance in meal preparation, house working, use of phone, use of transportation, shopping, managing finances, and taking medications (Landi et al., 2000). In previous papers, short‐ and long‐term effects of this randomized controlled trial (RCT) were reported (Corbett et al., 2015), as well as predictors of cognitive change in the GCT (Roheger et al., 2020a) and the ReaCT group (Roheger et al., 2020b).

The original study was approved by the St Thomas' Hospital Research Ethics Committee (Ref: 09/H0802/85) and registered on the International Standard Randomised Controlled Trial Number (ISRCTN) clinical trial database (Ref: ISRCTN72895114).

### 2.2 Participants

In total, data of *n* = 2912 participants were included in this post hoc analysis. Eligibility criteria were: (1) individuals older than 60 years and (2) access to a computer and the internet. Adults in the United Kingdom and abroad were invited to take part in this online RCT due to a partnership with the [Blinded for peer‐review]. Interested individuals were invited to register and consent to the study through a secure connection and ethically approved online process. In the following, participants received their login details and were randomized to a study group (GCT, ReaCT, or CG). To ensure that participants continued their training, weekly reminder Emails throughout the intervention were sent. A summary of performance and reinforcing text were automatically generated at the end of training sessions. Participants were included in the study when they participated in at least one training session.

### 2.3 Training interventions

GCT and ReaCT were investigated in comparison to a CG in the original study. Participants were asked to undertake the online training for at least 10 minutes daily, although flexibility was allowed. Training time was not tracked. Only the number of completed sessions per participant was recorded as an integrated feature in the online platform. GCT focused on six CT tasks covering mathematics, attention, memory and visuospatial abilities, ReaCT included three reasoning and three problem‐solving training tasks. An overview and more details on the training tasks are displayed in Table 1. Throughout the training, task difficulty increased as participants improved. The CG performed internet‐based tasks (e.g., a game in which people were asked to put a series of statements in correct numerical order with the help of internet searches).

**TABLE 1:** Training sessions included in the ReaCT and GCT groups delivered to respective treatment groups

Training session	Task	Main outcome measure
*General cognitive training*		
Attention 1	Click on rapidly appearing symbols as quickly as possible, but only if it matched one of the “target” symbols presented at the top of the screen.	Total number of correct trials across the two runs.
Attention 2	Select numbers in order from the lowest to the highest from a series of slowly moving, rotating, numbers.	Total number of correct trials across the two runs.
Memory 1	State the number of remaining items of baggage left in an airport x‐ray machine after watching a sequence of items moving down a conveyer belt toward the machine. The number of bags going in did not equal the number of bags coming out.	Number of problems completed in 3 min.
Memory 2	Identify matching pairs of picture cards after being shown the images and the cards being flipped over.	Total number of correct trials across the two runs.
Maths	Complete simple math sums (e.g., 17 −9) as quickly as possible.	Total number of correct trials across the two runs.
Visuospatial	Find the missing piece from a jigsaw puzzle by selecting from six alternatives.	Total number of correct trials across the two runs.
*Reasoning cognitive training*		
Reasoning 1	Use weight relationships, implied by the position of two seesaws with objects at each end, to select the heaviest object from a choice of three.	Total number of correct trials across the two runs.
Reasoning 2	Select the “odd one out” from four shapes that varied in terms of color, shape, and solidity (filled/unfilled).	Total number of correct trials across the two runs.
Reasoning 3	Move crates from a pile, each move being made with reference to the effect that it would have on the overall pattern of crates and how the result would affect future moves.	Total number of correct trials across the two runs.
Planning 1	Draw a single continuous line around a grid, planning ahead such that current moves did not hinder later moves.	Number of problems completed in min.
Planning 2	Move objects around between three jars until their positions matched a “goal” arrangement of objects in three reference jars.	Total number of correct trials across the two runs.
Planning 3	Slide numbered “tiles” around on a grid to arrange them into the correct numerical order.	Number of problems completed in 3 min.

*Note*. This table was taken and modified from Corbett et al. (2015). All sessions consisted of two 90 s “runs.”

### 2.4 Outcome measures

The primary outcome in the original study was self‐reported IADL at baseline, 6 weeks, 3 months, and 6 months. The Minimum Data Set‐Home Care IADL scale was used to measure IADL, as this scale has been extensively used in healthy older adults (Teresi & Holmes, 1997; Willis et al., 2006). IADL data were collected only in participants older than 60 following consultation with patient representatives and an ethics panel, who advised that the content of an IADL scale would not be acceptable to younger participants. The IADL scale is based on four levels of self‐performance in meal preparation, house working, use of phone, use of transportation, shopping, managing finances, and taking medications (Landi et al., 2000). Each of these IADL categories is further divided in subscale A and B for coding. Scale A reports the independence of the participants (ranging from 0 points “activity could be done independently” to 3 points “activity is done by another person”), Scale B reports the difficulty of the participants doing these activities (ranging from 0 points “no difficulty” to 2 points “great difficulty”). Therefore, for both subscales, higher scores indicate stronger impairment. The IADL score is the sum of the above items, thus IADL A scores ranged from 0 to 21 points, and IADL B scores ranged from 0 to 14 points (Landi et al., 2000).

### 2.5 Predictors

All variables assessed in the study that could possibly predict CT responsiveness on IADL were included, that is, age (numerical variable, in years), sex (male vs. female), education (categorized in five categories: none, primary school, secondary school, further education, university graduate), group (either ReaCT vs. CT or GCT vs. CT), level of depressive symptoms (assessed as a numerical variable on the Patient Health Questionnaire (Kroenke et al., 2001)), time, and number of intervention sessions. The Patient Health Questionnaire is a multiple‐choice self‐report inventory that is used as a screening tool for mental health disorders. The time variable was coded continuously for days. The number of training sessions was assessed as the total number of training sessions a participant completed until the time of measurement (as a continuous variable). Predictor assessment was blinded due to the online study design.

### 2.6 Statistical analysis

To explore the course of our outcome variables IADL part A and B, and to detect possible predictors for participants’ performance on IADL after CT, multilevel models were calculated using the *nlme* R package (R Core Team, 2020). Multilevel models, which can be described as linear mixed effect models that focus on research designs in which random effects are nested (Longford et al., 1993), have notable statistical advantages for measuring change compared to general linear models: optimal treatment of heterogeneity in retest schedules that randomly vary across participants, the ability to easily accommodate missing data, a statistical integration across levels of analysis that incorporates dependencies among observations both between and within individuals, and flexible assumptions about covariance across measurements (Hox et al., 2018). Finding the right model for the course of IADL performance over time is a prerequisite for the calculation of predictors of this performance. Therefore, we calculated random intercept models and random slope models for both outcomes (IADL A, IADL B) for each training (GCT and ReaCT, compared to CG) and tested with a likelihood ratio test to explore the course of IADL functioning to decide which model fitted better to the corresponding data. In case of a better fit of the random slope model, we also calculated a random slope and random intercept model and tested with a likelihood ratio test which model fits best. In the second step, we integrated the predictors in the best fitting model for each outcome. Time was coded continuously in days (1, 42, 84, 168 days). However, due to the real‐life setting of the assessment, the day of outcome completion may vary in several participants in a range of 48 h around these time points. All mentioned predictors were integrated with the multilevel models for the outcomes in two steps. First, all predictors were integrated solely; in the second step, all predictors and their interaction with the time variable were assessed (Mattes & Roheger, 2020). Further details can be obtained in Statistical Appendix of the Supporting Information.

## 3 RESULTS

### 3.1 Demographic characteristics of the sample at all three measurements

**FIGURE 1:**
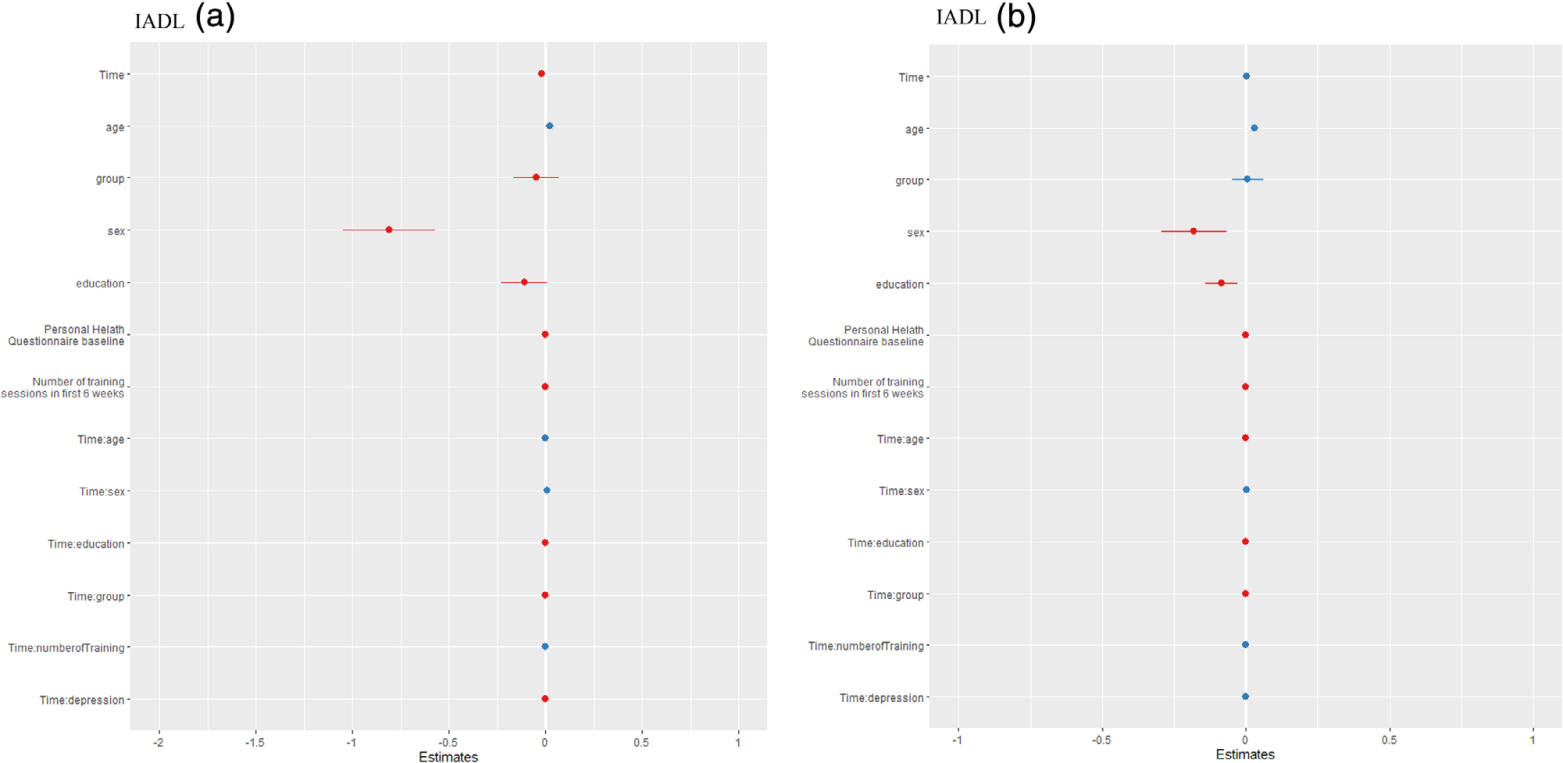
Multilevel models of instrumental activities of daily living parts A and B for reasoning cognitive training

1096 participants (*n *= 1920 female, *n *= 992 male) were included in the GCT, *n *= 1022 participants were included in the ReaCT group, 794 participants were included in the CG at baseline. Baseline IADL A performance ranged from 0−20 points with mean scores of 0.86 (*SD *= 2.67), indicating independence in about one of the investigated domains, but high heterogeneity. IADL B performance at baseline ranged from 0 to 14 points, with a mean IADL B score of 0.50 (*SD *= 1.25), indicating minor difficulties in the performance of one of the assessed IADL tasks, but again with high heterogeneity. Our sample, therefore, represents a group of older participants who are largely fit in IADL independence and difficulty, but do show heterogeneity with some individuals having marked impairment in IADL functioning. Cognitive scores showed no cognitive impairment as well as no baseline group differences. A flow chart of the participants throughout the study is displayed in Figure 1. Table 2 presents the demographic characteristics of all groups for participants older than 60 years at all time points.

**TABLE 2:** Descriptive statistics of all three groups at baseline, 6 week follow‐up, 3 months and 6 months follow‐up for participants older than 60 years

	Baseline (*n* = 2912)				6 week follow‐up (*n* = 2,809)				3 month follow‐up (*n* = 1620)				6 month follow‐up (*n* = 608)			
Characteristics	GCT *n* = 1096	ReaCT *n* = 1022	Control *n* = 794	*p*‐value	GCT *n* = 1062	ReaCT *n* = 1004	Control *n* = 743	*p*‐value	GCT *n* = 622	ReaCT *n* = 651	Control *n* = 347	*p*‐value	GCT *n* = 245	ReaCT *n* = 271	Control *n* = 92	*p*‐Value
Age, years	64.8 (4.6)	64.9 (4.9)	65.0 (4.9)	.706	64.84(4.61)	64.95(4.94)	64.89(4.84)	.791	64.86 (4.48)	64.73 (4.68)	65.52 (5.20)	<.05	64.83 (4.39)	64.85 (4.69)	65.82 (5.32)	.183
Sex, female	67.20	68.20	61.30	<.05	67.80	68.00	61.50	<.05	68.60	70.70	59.70	<.05	74.30	70.50	53.30	<.05
Education				.456				.610				.647				.151
None	2.70	3.00	2.50		2.60	2.90	2.60		1.90	2.50	1.40		1.60	1.80	1.10	
Primary school	0.50	0.90	0.90		0.50	0.90	0.90		0.30	0.80	0.60		0.40	1.10	1.10	
Secondary school	19.00	17.30	20.20		18.60	17.20	19.90		18.00	16.10	17.30		20.00	14.40	22.80	
Further education	30.60	29.10	32.20		30.70	29.40	31.60		31.70	29.60	34.60		28.20	29.20	39.10	
University graduate	47.20	49.70	44.20		47.60	49.60	45.00		48.10	51.00	46.10		49.80	53.50	35.90	
IADL																
Part A	0.82 (2.6)	0.93 (2.7)	0.85 (2.7)	.629	0.83 (2.67)	0.94 (2.78)	0.83 (2.67)	.488	0.79 (2.75)	0.92 (2.87)	0.92 (2.93)	.631	0.88 (2.43)	1.02 (3.06)	1.09 (2.49)	.789
Part B	0.53 (1.2)	0.50 (1.2)	0.53 (1.3)	.843	0.52 (1.23)	0.50 (1.18)	0.52 (1.36)	.899	0.55 (1.30)	0.52 (1.24)	0.54 (1.38)	.941	0.59 (1.39)	0.61 (1.46)	0.96 (1.72)	.103
Cognitive tasks																
Reasoning test	13.21 (5.3)	13.46 (5.3)	13.28 (5.1)	.536	13.23 (5.31)	13.43 (5.33)	13.26 (5.05)	.625	13.25 (5.23)	13.40 (5.33)	13.13 (4.76)	.713	13.19 (5.49)	13.60 (5.34)	13.48 (4.48)	.678
SWM Test	4.83 (1.20)	4.95 (1.20)	4.92 (1.12)	.055	4.82 (1.21)	4.95 (1.20)	4.93 (1.27)	<.05	4.88 (1.20)	4.94 (1.25)	4.95 (1.16)	.577	4.92 (1.17)	5.06 (1.17)	4.98 (1.17)	.353
Paired learning	3.41 (0.62)	3.42 (0.59)	3.42 (0.58)	.915	3.42 (0.61)	3.42 (0.59)	3.43 (0.59)	.778	3.41 (0.62)	3.42 (0.59)	3.41 (0.57)	.874	3.41 (0.58)	3.40 (0.64)	3.36 (0.60)	.872
Digit span	4.59 (1.11)	4.62 (1.13)	4.53 (1.14)	.239	4.59 (1.11)	4.62 (1.13)	4.53 (1.14)	.307	4.54 (1.15)	4.66 (1.13)	4.52 (1.18)	.102	4.49 (1.23)	4.75 (1.06)	4.48 (1.13)	<.05

*Note*: Age (in years), Baddeley Grammatic Reasoning Test, SWM Test. Paired Associate Learning Test, Digit Span Ladder Test, and IADL Part A and B are reported with means and standard deviations. All other values are *n* (%).

*p*‐values indicate group differences between the three groups at each of the four time points. Group differences were calculated using ANOVAs and Chi‐square tests, where appropriate.

*Abbreviations*: ReaCT, Reasoning Cognitive Training; GCT, General Cognitive Training.

### 3.2 What is the course of IADL functioning over time?

The first research question was whether our IADL outcome measures do or do not vary across individuals. While in a former paper, data were analyzed answering the question whether overall participants show better IADL performance after 6 weeks, 3 months, and 6 months of either GCT or ReaCT (Corbett et al., 2015), here we investigate the overall course of performance, indicating whether individuals showed different change rates over time. Results indicate that the random slopes models fitted better for the outcomes IADL Part B in the GCT group (χ^2^(2) = 18.78, *p* < .01), and both IADL Part A and Part B in the ReaCT group (χ^2^(2) = 28.57, *p* < .01; χ^2^(2) = 63.38, *p* < .01, respectively) indicating that every individual has a different change rate in his or her performance. Yet, for the outcome IADL Part A in the GCT group, the random intercept model had a better fit to describe the data (IADL A, GCT: χ^2^(2) = 0.02, *p* = .989), indicating that participants do vary in their intercepts, but not in their slopes/change rate over time. Results of the random slope model are depicted in Table 3, and results of the random intercept models in Table S1.

**TABLE 3:** Fixed and random effects of the outcome variables as a function of time in a random slope model

Parameter	Coefficient	*SE*	*T*‐value	*df*	*p*	*AIC*
**Fixed effects: General cognitive training**						
IADL Part A						22,010.23
Intercept	0.83	0.06	15.06	3111	<.01	
Time slope	−0.01	0.00	−2.15	3111	<.05	
IADL Part B						13,729.07
Intercept	0.52	0.3	18.69	3111	<.01	
Time Slope	−0.01	0.00	−6.26	3111	<.01	
**Fixed effects: Reasoning cognitive training**						
IADL Part A						22,319.01
Intercept	0.86	0.06	14.78	3107	<.01	
Time slope	−0.01	0.00	−2.23	3107	<.05	
IADL Part B						13,212.13
Intercept	0.49	0.03	17.80	3108	<.01	
Time slope	−0.01	0.00	−4.97	3108	<.01	
	*SD*	Correlation		*SD*	Correlation	
**Random effects: General cognitive training**				**Random effects: Reasoning cognitive training**		
IADL Part A				IADL Part A		
Time slope	0.00	−0.02	Time Slope	0.01	−0.58	
Level 1 residual	1.64		Level 1 residual	1.83		
IADL Part B			IADL Part B			
Time slope	0.00	−0.20	Time Slope	0.01	−.24	
Level 1 residual	0.62		Level 1 residual	0.59		

*Abbreviations*: df, degrees of freedom; SD, standard deviation; SE, standard error.

### 3.3 What are predictors for individual differences in the outcome variables?

To test what variables predict individual differences in the outcome variables, we first calculated models in which the predictors were tested solely (for the results see Table S2), but only for the random slopes models, that is, IADL part A and B of ReaCT, and IADL part B for GCT, as we wanted to detect predictors for changes over time and not for changes in the intercept. In a second step, all predictors with their interaction with the time variable were tested; results are depicted in Table 4 and Figure 1. Significant Time*Predictor interactions could be obtained in IADL Part A and B of the ReaCT, showing female sex as a positive predictor for lower IADL values (i.e., better IADL performance) over time. No other significant predictors were observed.

**TABLE 4:** Fixed effects of the outcome variables and predictors for individual differences

Predictors	Outcomes			
	General cognitive training		Reasoning cognitive training	
	IADL Part A	IADL Part B	IADL Part A	IADL Part B
*Fixed effects*	*Coefficient (SE)*	*Coefficient (SE)*	*Coefficient (SE)*	*Coefficient (SE)*
Intercept	0.24 (0.88)	−0.89 (0.44)*	1.37 (0.88)	−0.76 (0.41)
Time	−0.01 (0.01)	0.00 (0.00)	−0.02 (0.01)*	0.00 (0.00)
Age	0.03 (0.01)**	0.03 (0.01)***	0.02 (0.01)	0.03 (0.01)***
Group	−0.01 (0.11)	−0.02 (0.06)	−0.04 (0.06)	0.01 (0.28)
Sex	−0.65 (0.11)***	−0.17 (0.06)**	−0.81 (0.12)***	−0.18 (0.06)**
Education	−0.09 (0.06)	−0.09 (0.03)**	−0.11 (0.06)	−0.08 (0.03)**
Severity of depression	−0.00 (0.00)	−0.00 (0.00)	−0.00 (0.00)	−0.00 (0.00)
No. of trainings	−0.00 (0.00)	0.00 (0.00)	−0.00 (0.00)	−0.00 (0.00)
Time*Age	0.00 (0.00)	−0.00 (0.00)	0.00 (0.00)	−0.00 (0.00)
Time*Group	−0.00 (0.00)	0.00 (0.00)	−0.00 (0.00)	‐0.00 (0.00)
Time*Sex	0.01 (0.01)***	0.00 (0.00)	0.01 (0.00)***	0.00 (0.00)**
Time*Education	−0.00 (0.00)	−0.00 (0.00)	−0.00 (0.00)	−0.00 (0.00)
Time*Severity of depression	0.00 (0.00)	0.00 (0.00)	−0.00 (0.00)	0.00 (0.00)
Time*No. of trainings	0.00 (0.00)	−0.00 (0.00)	0.00 (0.00)	0.00 (0.00)
AIC/BIC	21,971.46/22,088.77	13,688.99/13,806.30	22,276.56/22,393.59	13,173.82/13,290.85

*Abbreviations*: SE, standard error.

Significant values:

* < .05,

** < .01,

*** < .001.

## 4 DISCUSSION

This is the first paper investigating predictors of IADL changes in the long term during CT. Our multilevel analysis including *n *= 2912 adults older than 60 years receiving GCT or ReaCT or no intervention investigated two different IADL outcome variables: independence (IADL part A) and difficulty of IADL tasks (IADL part B). Results in our sample which showed an average mild IADL changes show that (i) the course of IADL functioning of the ReaCT group for both outcomes and of the GCT group for the IADL part A can be best described as a random sloped model, indicating different changes over time for individual participants, (ii) female sex is a significant predictor for improvement in IADL Part A and Part B, but only after ReaCT, while no other significant predictors were found.

Our results show that IADL performance curves do vary across individuals over time when receiving GCT and ReaCT, except for the IADL part A outcome of the GCT indicating that IADL performance during a phase in which CT is conducted is influenced by different individual factors. Female sex seems to be the most influential individual factor for IADL performance differences in the ReaCT group. Remarkably, a cross‐national comparison of sex differences in IADL in Europe in 51,292 men and 62,007 women aged 50+ conducted by Scheel‐Hincke et al., in 2020 revealed that women had a higher risk than men in IADL limitations and that sex differences increase even with advancing age (Scheel‐Hincke et al., 2020). As female sex was a predictor for improvement in IADL over time in our study, it may be possible that the compensation account explains this pattern (Lövdén et al., 2012), even though in our mildly affected sample no significant baseline differences in IADL performance were detected. This account implies that individuals who are already functioning at optimal levels have less room for improvement in performance, whereas those with low function may improve to a greater degree. Our results may have important implications, as CT might be an effective and also home‐based, easy to implement intervention to stabilize IADL function – especially in women. Notably, even though data on sex difference is still rare in the field of prediction of changes after non‐pharmacological interventions, we could also show in a previous analysis of data from the same RCT that being female was a predictor for changes in different cognitive domains in the same population (Roheger et al., 2020; Roheger et al., 2020). Yet, it is important to be aware that, in general, investigated sex differences often have small effect sizes and further research is urgently needed (Choleris et al., 2018).

Strikingly, results showed female sex as a significant predictor only for the ReaCT, but not for GCT. A study by Willis et al. (2006) also showed stronger effects of ReaCT on IADL than GCT in the “ACTIVE” trial: they trained a sample of *n* = 2832 older participants in either memory or reasoning or speed of processing training and could show that only the reasoning and the speed of processing training showed less self‐reported IADL decline—5 years after the actual training (Willis et al., 2006). This may be explained by the fact that ReaCT more strongly trains executive functions and working memory capacities, which are shown to be strongly correlated with IADL performance (Choleris et al., 2018). Several IADL tasks as, for example, handling finances require executive functions such as planning and monitoring—therefore, executive or reasoning training might be especially suited to improve IADL tasks. Yet, the ACTIVE study did not investigate and report any sex‐dependent effects and to the author's best knowledge, no studies exist specifically investigating sex differences in effects of reasoning training on IADL performance. Interestingly, in regard to our findings, it is important to consider that IADL tasks may be sex‐stereotyped in an elderly cohort. Feger et al. (2020) pointed out, for example, that women traditionally perform IADL activities such as cooking and cleaning‐related tasks, whereas men traditionally handle finances and this may influence performance in IADL scales. Therefore, the developers of early IADL scales suggested using differential scoring for males and females (Lawton & Brody, 1969). Also, reports of IADL difficulty and performance across birth cohorts have shown that the younger cohorts of older adults are less likely to show these stereotyped behaviours (Sheehan et al., 2019). Thus, it is also possible that our results may be biased by these sex‐stereotypes underlying traditional IADL performances, as we did not use two separate IADL measures for men and women. However, it is important to notice that trends in IADL performance across birth cohorts have shown that the younger cohorts of older adults are less likely to exhibit sex‐stereotyped behaviours as Sheehan et al. (2019) found out in a cohort study conducted in the United States.

Surprisingly, no other of our investigated predictors (namely age, education, level of depressive symptoms, time, and number of intervention sessions) predicted changes in IADL performance. This is rather unexpected, for example, IADL disabilities highly correlate with neuropsychological functioning and, therefore, are more sensitive to cognitive change due to aging (Kim et al., 2021). Yet, it may be possible that our sample in the present study was still too cognitively healthy to show specific cognitive and also IADL impairments, explaining why age and potentially also education (as a proxy for cognitive development in aging (Krieger et al., 1997)) was not a significant predictor. Also, as we were investigating participants with beginning and/or mild IADL changes, the levels of depressive symptoms may not be high enough to have a significant impact on our results.

Identifying prognostic factors is highly important for providing new and personally tailored treatment options and in terms of dementia prevention (Riley et al., 2013). Therefore, several studies have investigated individual predictors of CT success for improving cognition and cognitive variables (Roheger et al., 2020; Roheger et al., 2020), however, to the author's best knowledge, this is the first study investigating predictors of IADL performance in older adults, even though maintaining functional independence is of great importance for older adults. One particular strength of the present paper is that IADL performance was measured across a period of 6 months, allowing to identify longitudinal predictors that exhibit reliable change over a specific interval. Generally, collecting longitudinal as opposed to cross‐sectional estimates of predictor variables permits the examination of the dynamic interaction between change in possible predictor variables and change in outcome variables. Future predictor studies should also use a longitudinal design with several measurement points to estimate the changes over time. A further strength is the large sample that allows for the inclusion of multiple predictors in our analysis. Yet, a possible limitation is that the sample may be biased due to the fact that often highly educated and highly motivated participants conduct cognitive trainings (Schubert et al., 2014), although this is a more general problem of CT studies. Therefore, the generalizability of the results may be limited to highly educated and motivated participants with only mild IADL changes. Future research needs to be conducted in a more impaired and even broader sample regarding sociodemographic variables.

## 5 CONCLUSIONS AND IMPLICATIONS

To summarize, female sex predicted improvements in IADL in cognitively fit healthy older participants with beginning or mild IADL changes (e.g., minor, not clinical dysfunction in some but not all measured IADL) during a 6 month ReaCT indicates that females had a greater change in IADL performance compared to men. Yet, as previous results of our study showed, both investigated trainings are effective in improving IADL performance (Corbett et al., 2015). Maintaining functional independence is of great importance for older adults as it is associated with increased quality of life, and also decreased IADL capacities are strongly correlated with development of cognitive decline and dementia. Further research should unravel prediction patterns and underlying mechanisms of improvements after non‐pharmacological interventions to tailor them to individuals with different profiles—also in terms of dementia prevention. One particular focus should not only lay on cognitive outcomes, but also on IADL, mood‐related variables, and quality of life.

## CONFLICT OF INTEREST

The authors declare no conflict of interest.

### PEER REVIEW

The peer review history for this article is available at https://publons.com/publon/10.1002/brb3.2388

## Supporting information

Supporting information
