# Supplementary material for: Predictors of activities of daily living in heathy older adults: Who benefits most from online cognitive training?
Source: Brain Behav. 2021 Oct 17;11(11):e2388. doi: 10.1002/brb3.2388 (PMC8613408; doi:10.1002/brb3.2388)
Supplement: Supplementary file 1 — Supporting information [file BRB3-11-e2388-s001.docx]

Supplementary Material:

Predictors of activities of daily living in heathy older adults: who benefits most from online cognitive training?

**Statistical Appendix**

The initial research question tested whether our outcome measure does or does not vary across individuals with the help of multilevel modeling. Therefore, in the first step, random intercept models were calculated, where there are no variations in the slopes of the model, but the intercepts can differ between individuals. A random intercept model in which an individual’s response depends linearly on time can be written:

1. $y_{ij}= \beta_{0}+\upsilon_{0j}+\beta_{1j}t_{ij}+e_{ij}$

$\beta_{0}$ is defined as the overall intercept (averaged across individuals) and $\beta_{1}$ is the slope of the regression of $y$ on time, commonly referred to as the growth rate. The growth rate is assumed to be the same for all individuals in a random intercept model. The models are fit with the log-likelihood method. The Time variable is coded continuously in days. Results of the random intercept models for each outcome are displayed in Supplementary Material Table 1.

A random slope model was conducted, which allows for variation between individuals in the rate of change of our dependent variable, as well as in their level of the dependent variable at any occasion (the intercept). The single-equation form of the random slope model can be written as:

$$\left( 2 \right) y_{ij}= \beta_{0}+\beta_{1}t_{ij}+\upsilon_{0j}+\upsilon_{1j}t_{ij}+e_{ij}$$

Individual variation in the slope is indicated by the *j* subscript on the slope parameter *β_1._*  The growth rate for individual *j* is now equal to the overall average slope *β_1_* (common to all individuals) plus a random amount $\upsilon_{1j}$ specific to individual *j*. The two individual-level random effects $\upsilon_{0j}$ and $\upsilon_{1j}$ are assumed to follow a bivariate normal distribution with zero mean. Results of the random slope models are displayed in the Manuscript, Table 3. The random intercept models and the random slope models were compared for each outcome variable with a log-likelihood test to see which model has the better fit to the data.

| Parameter | Coefficient | *SE* | T-value | *df* | *p* |
| --- | --- | --- | --- | --- | --- |
| **Fixed effects: General Cognitive Training** | | | | | |
| IADL Part A |  |  |  |  |  |
| Intercept | 0.82 | 0.06 | 15.06 | 3,111 | <.01 |
| Time | -0.01 | 0.00 | -2.15 | 3,111 | <.05 |
| IADL Part B |  |  |  |  |  |
| Intercept | 0.52 | 0.03 | 18.9 | 3,111 | <.01 |
| Time | -0.01 | 0.00 | -6.77 | 3,111 | <.01 |
| **Fixed effects: Reasoning Cognitive Training** | | | | | |
| IADL Part A |  |  |  |  |  |
| Intercept | 0.87 | 0.06 | 15.56 | 3,107 | <.01 |
| Time | -0.00 | 0.00 | -2.35 | 3,107 | <.05 |
| IADL Part B |  |  |  |  |  |
| Intercept | 0.49 | 0.03 | 18.02 | 3,108 | <.01 |
| Time | -0.01 | 0.00 | -5.43 | 3,108 | <.01 |
|  | *SD* |  |  | *SD* |  |
| **Random effects: General Cognitive Training** | | | **Random effects: Reasoning Cognitive Training** | | |
| IADL Part A | | | IADL Part A | | |
| Intercept | 1.93 |  | Intercept | 1.74 |  |
| Residual | 1.64 |  | Residual | 1.88 |  |
| IADL Part B |  |  | IADL Part B |  |  |
| Intercept | 1.05 |  | Intercept | 1.03 |  |
| Residual | 0.65 |  | Residual | 0.64 |  |

Table 1: Fixed and Random Effects of the Outcome Variables as a Function of Time in a Random Intercept model

Table 2: Fixed Effects of the Outcome Variables and Predictors for Individual Differences

| **Predictors** | **Outcomes** | | | |
| --- | --- | --- | --- | --- |
|  | General Cognitive Training | | Reasoning Cognitive Training | |
|  | IADL Part A | IADL Part B | IADL Part A^1^ | IADL Part B |
| *Fixed Effects* | *Coefficient (SE)* | *Coefficient (SE)* | *Coefficient (SE)* | *Coefficient (SE)* |
| Intercept | -0.26 (0.81) | -0.71(0.42) | - | -0.68(0.38) |
| Time | -0.00(0.00)* | -0.00(0.00)*** | - | -0.00(0.00)*** |
| Age | 0.04(0.01)*** | 0.03(0.01)*** | - | 0.02(0.01)*** |
| Group | -0.05(0.10) | -0.03(0.05) | - | 0.00(0.03) |
| Sex | -0.44(0.11)*** | -0.13(0.05)* | - | -0.12(0.05)** |
| Education | -0.11(0.05)* | -0.10(0.03)*** | - | -0.09(0.02)*** |
| Severity of depression | -0.00(0.00) | -0.00(0.00) | - | -0.00(0.00) |
| No. of trainings | -0.00(0.00) | -0.00(0.00) | - | 0.00(0.00) |
| AIC / BIC | 21,983.41/  22,061.62 | 13,687.68/  13,765.89 | - | 13,176.15/  13,254.18 |

*Note. Abbreviations*: SE = Standard error. Significant values: * < .05, ** < .01, *** < .001, ^1^ Model does not converge.
